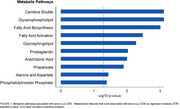# Metabolome Wide Association Study of Serum DDE in Midlife Adults: Relevance to Prodromal ADRD

**DOI:** 10.1002/alz70855_106825

**Published:** 2025-12-24

**Authors:** Carolyn Accardi, Mary Nellis, Bill Liang, ViLinh Tran, Young‐Mi Go, Dean P Jones, Piera Cirillo, Nickilou Krigbaum, Pam Factor‐Litvak, Isha Mhatre‐Winters, Barbara Cohn, Jason R Richardson

**Affiliations:** ^1^ Emory University School of Medicine, Atlanta, GA, USA; ^2^ Child Health and Development Studies, Public Health Institute, Oakland, CA, USA; ^3^ Columbia University Mailman School of Public Health, New York, NY, USA; ^4^ Isakson Center for Neurological Disease Research, College of Veterinary Medicine, University of Georgia, Athens, GA, USA

## Abstract

**Background:**

Serum levels of dichlorodiphenyldichloroethylene (DDE), the primary metabolite of the organochlorine pesticide dichlorodiphenyltrichloroethane (DDT), are associated with cognitive impairment and increased risk of Alzheimer's Disease (AD). High‐resolution metabolomics (HRM) studies have demonstrated metabolic phenotypes associated with early cognitive changes and AD risk with specific metabolome perturbations in amino acid and fatty acid metabolism. Metabolome changes associated with serum *p,p’‐*DDE in midlife that may precede cognitive dysfunction and AD are yet to be characterized.

**Method:**

This study analyzed subjects from a prospective 60‐yr follow up study of the Child Health and Development Studies (CHDS), which evaluates developmental origins of health including exposure to environmental toxicants. A cross‐sectional analysis of 362 subjects (172 females/190 males, ages 44.9‐52.0 yrs, mean 46.8 yr) measured midlife serum *p,p’*‐DDE simultaneously with the serum metabolome and evaluated *p,p’*‐DDE‐associated metabolic responses. HRM was performed using liquid chromatography/mass spectrometry (LC‐MS) (HILIC/ESI +/C18 ESI‐ ionization modes) for the serum metabolome and gas chromatography/mass spectrometry (GC‐MS) for the serum exposome and *p,p’*‐DDE measures. A metabolome‐wide association study (MWAS) conducted on the untargeted LC‐MS data identified specific pathways associated with serum *p,p’*‐DDE using regression analysis and *mummichog* pathway analysis.

**Result:**

Multiple metabolic pathways involved in fatty acid metabolism were associated with *p,p’*‐DDE, including the carnitine shuttle, fatty acid biosynthesis, and arachidonic acid, prostaglandin, and glycerophospholipid metabolism. Metabolism of the amino acids alanine and aspartate were also linked to *p,p’*‐DDE (Figure 1). Several saturated, monounsaturated, and polyunsaturated fatty acids decreased with increasing serum *p,p’*‐DDE, including the essential fatty acids docosahexaenoic acid (DHA)(adjusted *p*‐value =0.004) and eicosapentaenoic acid (EPA)(adjusted *p*‐value =0.006). We also found a decrease in acylcarnitines with increasing serum *p,p’*‐DDE. The metabolic profiles replicate previous findings of metabolic changes implicated in cognitive decline and AD, including declines in plasma DHA, fatty acid perturbations, and mitochondrial metabolic dysfunction.

**Conclusion:**

The DDE‐associated metabolic changes in midlife overlap those previously associated with DDE, DDT and with cognitive dysfunction and AD diagnosis. Thus, relating midlife serum DDE and metabolome to cognitive function will elucidate important metabolic features and biological mediators in cognitive decline and AD onset in adults.